# Worthless and Nutritive Nuptial Gifts: Mating Duration, Sperm Stored and Potential Female Decisions in Spiders

**DOI:** 10.1371/journal.pone.0129453

**Published:** 2015-06-24

**Authors:** Maria J. Albo, Alfredo V. Peretti

**Affiliations:** 1 Laboratorio de Etología, Ecología y Evolución, IIBCE, Montevideo, Uruguay; 2 Instituto de Diversidad y Ecología Animal, CONICET-, Universidad Nacional de Córdoba, Córdoba, Argentina; UC Santa Barbara, UNITED STATES

## Abstract

In nuptial gift-giving species females sometimes select their potential mates based on the presence and size of the gift. But in some species, such as the Neotropical polyandrous spider *Paratrechalea ornate* male gifts vary in quality, from nutritive to worthless, and this male strategy can be in conflict with female nutritional benefits. In this species, males without gifts experience a reduction in mating success and duration, while males that offer worthless or genuine nutritive gifts mate with similar frequencies and durations. The female apparently controls the duration of copulation. Thus, there is scope for females to favour males offering gifts and further if these are nutritious, via post-copulatory processes. We first tested whether females differentially store sperm from males that offer the highest nutritional benefits by experimentally presenting females with males that offer either nutritive or worthless gifts (uninterrupted matings). Second, we carried out another set of experiments to examine whether females can select sperm based only on gift presence. This time we interrupted matings after the first pedipalp insertion, thus matching number of insertions and mating duration for males that: offered and did not offer gift. Our results showed that the amount of sperm stored is positive related to mating duration in all groups, except in matings with worthless gifts. Gift presence itself did not affect the sperm stored by females, while they store similar number of sperm in matings with males offering either nutritive or worthless gifts. We discuss whether females prefer males with gifts regardless, if content, because it represents an attractive and/or reliable signal. Or alternatively, they prefer nutritive nuptial gifts, as they are an important source of food supply and/or signal of male donor ability.

## Introduction

Processes of inter and intra-sexual selection have enormous potential to shape behavioural, morphological and physiological traits involved in securing copulations and fertilization. These selective pressures can act on male and female traits before (pre-copulatory), during (syn-copulatory) or after mating (post-copulatory) [1–3]. Cryptic syn- or post-copulatory sexual selection occurs internally in polyandrous females, and can comprise sperm competition [3] as well as cryptic female choice of sperm [2, 4–9] both responsible for biasing male paternity. While sperm competition involves males´ gamete competition inside the female, the idea of cryptic female choice refers to any mechanism performed by females to select males´ gametes [2]. These internal processes have been far complex to study because they not only can be shaped by either sex but also by interactions between partners, as was recently discussed [10]. Nevertheless, it is known in many species that by adjusting mating duration females can determine the amount of sperm stored [6, 11–14]. There is also clear evidence that female decisions in fertilizing their eggs can be influenced by male copulatory courtship behaviours [2, 15, 16]. In many invertebrate species, it has been reported that males increase paternity success by rhythmically stimulating females [17–23]. This indicates that by evaluating male phenotype, females can gain critical information about males and favour those with the highest inherent quality [2, 24]. For instance, female red flour beetles can differentially store sperm depending on the male physical condition, as matings with well fed males result in more sperm stored than those with starved males [5].

Males from gift-giving species that offer an immediate nutritious meal to females may influence female post-copulatory decisions not only by providing direct benefits but also by signalling high genetic quality if the gift represents that they are good gift donors. In other words, the gift itself may represent food and good genes all at once [25, 26]. Thus, females should favour paternity from males with the ability to produce a nutritive and/or large gift. Those males would be ranked by females as better donors than males offering no gift or less valuable gifts. Indeed, there are two main mechanisms reported by which females select males in gift-giving mating systems: 1) by mating with males offering gifts during courtship, and 2) by modulating mating duration depending on gift size [27–30]. But, in some species males have evolved the ability to vary gift content from nutritive to worthless items, and offer both types of gifts [31–34]. It appears that females only perceive gift content after accepting a mating when they consume the gift. Thus, worthless gifts may be a source of conflict between sexes [35]. By offering worthless gifts males inexpensively maintain the advantage of transferring sperm. But, for females that engage in multiple matings to some extent because the foraging benefits of the gift [26] the worthless gift behaviour seems to operate against their nutritional interests. In this scenario, we would expect female mate choice to be exerted during and after mating, for instance by exercising control over the number of sperm stored depending on gift content and/or presence. Recently, it was reported that females of the spider *Pisaura mirabilis* cryptically select male´s sperm to fertilise the eggs based on gift presence [9]. Even though in gift-giving species, females have the potential to differentially store sperm from males that offer the largest nutritive benefits, there are still limited studies testing for cryptic female processes in these mating systems [10].

We investigated how sperm storage is related to mating duration, and whether females may differentially store sperm depending on gift content and presence in the Neotropical nuptial gift-giving spider *Paratrechalea ornata* (Trechaleidae). Females from this species are polyandrous [36] and favour matings and longer copulations with males offering nuptial gifts over males without gifts [37]. Virgin females are less selective, and sometimes accept matings with males that do not offer gifts [34]. However once mated, females radically become more reluctant to additional matings, even when a gift is offered [36]. Indeed, mated females exert such strong selection on gift-giving behaviour that males that have been rejected and do not have prey available usually collect and wrap inedible items or “worthless gifts” to obtain a mating [34]. In the field, 70% of the gifts carried by males are worthless (empty exoskeletons and/or plant parts) and 30% are nutritive gifts (fresh prey) [34]. These findings indicate a remarkable plasticity in gift-giving behaviour that differs among spider species. For instance, there are important differences in gift content compared with the Palearctic gift-giving spider *Pisaura mirabilis* (Pisauridae), in which nutritive gifts are the most common (70%) [33]. During pre-copulatory courtship, however, females from both species chose males based on gift presence and not content, as they accept matings with males offering worthless and nutritive gifts at similar frequency [33, 34]. This is not surprising, as females can only recognize gift content once they start to feed on it and the mating has begun. Since in *P*. *mirabilis* sperm stored in the female is positively correlated to mating duration [9], it is possible that females counteract male deception by shortening matings with males offering worthless gifts [33], consequently diminishing the number of sperm stored (Tuni C & MJ Albo unpublished data). In contrast, mating duration in *P*. *ornata* is similar between males offering nutritive and worthless gifts. As the mating is extremely short (1 min average) it can be possible that females may not have enough time to evaluate and recognize the gift content during copulation, alternatively they do not respond to it.

Two main hypotheses can be considered in order to understand how gift content may influence female decisions on sperm stored in *P*. *ornata*. Males offering worthless gifts are equally accepted as males with nutritive gifts by females, supporting the idea that gift presence per se correlates with male attractiveness and/or quality [25, 38]. If so, we would expect females not biasing their preferences via post-copulatory processes, for instance storing similar amounts of sperm, in matings with nutritive and worthless gifts. Alternatively, females favour nutritive gifts because these ones are food supply (contributing to female fecundity) and/or signal male donor ability. Under this hypothesis, we would expect females showing post-copulatory preference for nutritive gifts or males that give them. One way in which this could occur is to limit sperm stored via cryptic processes that results in less sperm when the gift is worthless, potentially affecting male paternity success. To examine whether gift content influences the number of sperm stored in the female spermathecae we carried out a set of experiments that included two uninterrupted mating groups: males offering nutritive gifts and males offering worthless gifts. On the other hand, besides prolonging the mating the gift presence itself can also influence sperm stored by females, as was shown in another spider [9]. If this is the case in *P*. *ornata*, we should expect that females mating with males offering gifts store more sperm than those mating with males lacking gifts at similar mating duration. Thus, we carried out another set of experiments to examine whether gift presence influences the number of sperm stored in the female spermathecae. This time we interrupted matings after the first insertion and therefore standardized the number of insertions and mating duration in males that offered gifts and males that did not offer gifts.

In this species, males usually performed long insertions of approximately more than 10 seconds [34; 37], however sometimes it has been observed that they can perform “short insertions” lasting less than 10 seconds. Short insertions have never been quantified and it is unclear whether they transfer sperm or not. Thus, we were also interested in quantify each type of insertion and examine how these can affect sperm stored in our experimental set ups.

## Materials and Methods

### Study species

We collected juveniles and subadults of *P*. *ornata* in August-September 2012 at Santa Lucía River (Paso del Molino, Arequita, Lavalleja, 34°16’40.10”S 55°14’00.80”W), Uruguay. This study did not involve endangered or protected species, and no specific permissions were required for the location and/or activity. In the laboratory, spiders were sexed and kept in plastic jars (8.5 cm inter diameter and 7.5 cm high) containing small pebbles. Water was provided regularly to maintain humidity. We raised individuals in a warm climate room 24.3°C (± 0.1 SE) to accelerate development, a procedure that is known to have no effects on spiders´ behaviours. Moults were checked daily and spiders were fed with fruitflies (*Drosophila melanogaster*) three times per week until maturation. Once females (N = 82) and males (N = 82) reached adulthood, we placed them in a room with an average temperature of 22.7°C (± 1.7 SE), and fed them with fruitflies twice per week. For all mating experiments and in order to control for any effect of individuals reproductive experience, we used virgin females and males and we did not reuse them. We measured adult size (cephalotorax width) in all individuals used in the experimental groups (N = 164) and to control for any size effect in the results. Adult male and female size differed slightly between groups. Adult male´s averaged size (mm ± SE) was: 3.7 ± 0.1 in males offering a Fly gift-uninterrupted mating; 4.0 ± 0.1 in males offering a Worthless gift-uninterrupted mating; 3.9 ± 0.1 in males offering a Fly gift-interrupted mating; 3.9 ± 0.1 in males offering No-fly gift-interrupted mating; while adult female´s averaged size (mm ± SE) was: 3.8 ± 0.1 in Fly and Worthless gift-uninterrupted mating; 4.0 ± 0.1 and 3.7 ± 0.1 in Fly and No-fly gift-interrupted mating, respectively.

### Mating behaviours and experimental design

Typically, the male wraps the gift in silk and offers it to the female in a particular posture called “hyperflexion” [39]. Female mating acceptance occurs when she grasps the gift with her chelicerae in a face-to-face position. As in other entelegyne spiders, both female and male genitalia are bilaterally symmetrical, males having two pairing intromittent organs (pedipalps) and females having two pairing copulatory openings, each one connected to a separate sperm storage organ (spermatheca) [40]. Thus, once the female accepts the gift, she allows the male to mount and initiate sperm transfer via pedipalp insertions into her genitalia. Males can perform up to four insertions during a mating. Between two insertions the male returns to a face-to-face position and again grasps the gift [37, 39]. Females stored the inactive and encapsulated sperm transferred by males, and later decapsulate and activate for egg fertilization [41, 42]. Mating behaviours are similar when no gift is present, including the time in the face-to-face position after pedipalp insertion [37].

### Gift content and sperm stored in the female spermathecae

To analyze whether gift content influences the number of sperm stored by females, we created two experimental groups (uninterrupted matings). The group Fly gift-uninterrupted mating included twenty-two males offering a fly gift (*Musca domestica*), and the group Worthless gift-uninterrupted mating included seventeen males offering a worthless gift (a dry exoskeleton from *Tenebrio molitor* larva, following the protocol from Albo and colleagues [34], see below. After mating, all females retained the gift and continued manipulating it.

### Gift presence and sperm stored in the female spermathecae

To examine whether the presence of a gift confers advantages to males in the number of sperm stored by females, we conducted another set of mating trials with two experimental groups where we interrupted the mating after one pedipalp insertion (interrupted matings). By doing this, we standardized mating duration in trials with and without a gift, since the gift presence itself prolongs copulation [37] and hence may affects sperm transfer. We interrupted matings by using a paintbrush, when the male returned to the face-to-face position after the first insertion. In the group Fly gift-interrupted mating nineteen males offering a fly gift were allowed to perform one pedipalp insertion for an average of 0.38 ± 0.04 min (mean ± SE). In the group No-fly gift-interrupted mating twenty-four males without fly gift were allowed to perform one pedipalp insertion for an average of 0.39 ± 0.07 min (mean ± SE). As expected, mating duration was similar between the two interrupted matings, Fly and No-fly gift (Student t-test: t_1,28_ = 0.50, p = 0.61, N _Fly gift-interrupted mating_ = 17, N _No-fly gift-interrupted mating_ = 13).

### Behavioural data

All mating experiments were carried out during October-December 2012. A virgin female was placed in a transparent plastic cage (30 cm diameter and 10 cm height) with pebbles covering the bottom one day before the experiment, allowing her to deposit silk threads which are important stimuli for male courtship [43]. We then exposed each female to one male carrying a wrapped housefly (Fly gift-interrupted and uninterrupted mating), or a wrapped exoskeleton (Worthless gift-uninterrupted mating), or no gift (No-fly gift-interrupted mating). As we wanted all males with gifts to wrap them (Fly and Worthless gifts), we elicited gift-wrapping by exposing the experimental males with a housefly or an exoskeleton to a mated female 30 min before the experiment started. These females were mated with another male in a different experiment. Mated females reject males more often than virgin ones [34, 36], and males usually wrap the item in silk after rejection [37]. We only allowed males to physically contact females once as we simulated female rejection by pushing her away with a paintbrush. Males without gift experienced the same procedure.

We registered the number of pedipalp insertions, whether the insertions were long or short and mating duration. We classified pedipalp insertions depending on their duration following Albo and colleagues [44]. ‘Long insertions’ lasted for at least 0.1 min while ‘short insertions’ were less than 0.1 min. Expansions of the hematodochae, a blood inflatable structure that allows injection of the sperm into the female genitalia [45], were observed for both long and short insertions. We registered the occurrence (yes/no) and number of long and short pedipalp insertions per mating and compared their frequencies between groups. Males can perform only long insertions, only short insertions or both types of insertions during a single mating. However, we wanted to know if females stored sperm from males that perform only short insertions or contrary, females stored sperm only from males performing long insertions. In both Fly and Worthless gift-uninterrupted mating, sperm number was similar whether males performed only long insertions (N = 15) or both long and short insertions (N = 17) during a mating (Student t-test: t_1, 30_ = 0.12, p = 0.90). Thus, if a mating contained both long and short insertions, we categorized it as with long insertions and compared it with matings containing only short insertions. On the other hand, in the Fly and No-fly gift-interrupted mating, we allowed males to perform only a single long insertion, but if they naturally performed short insertions, these last data were only used to compare the frequencies and the amount of sperm stored during both types of insertions. Mating duration was calculated as the sum of the duration of all long insertions occurring within a trial, from pedipalp insertion until pedipalp disengagement; because duration of short insertions was impossible to be measured these ones were not included in mating duration. Sperm count was done after matings and protocol details are given below.

### Sperm count

**Fig 1:**
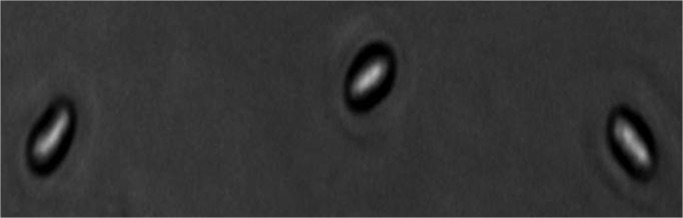
Picture of the capsulated sperm cells from *P*. *ornata* captured under a microscope (40x). Photo: MJ Albo.

Spiders have external digestion and they need to release digestive fluids necessary to absorb the prey. These fluids are sucked in and released into the prey while the tissue is gradually sucked out and the process of consuming the whole prey may take 1 hour or more [45]. In preliminary observations we registered that the time to consume the housefly gift varies between 1 and 4 hours. Thus, females from all groups were frozen 4 hours after mating at -80°C, giving them time to consume the whole gift in the case of the Fly gift-interrupted and uninterrupted mating and controlling for the same time in the case of No-fly gift-interrupted mating and Worthless gifts-uninterrupted mating. As female´ spiders stored encapsulated sperm and decapsulation takes place several days after mating [41, 42], we did not find decapsulated sperm in our samples and the sperm counts were based on encapsulated sperm (Fig 1). For counting the number of sperm stored, both female spermathecae were dissected out under a stereomicroscope (Olympus, SZH) and treated following a protocol established for spiders [46]. We transferred both female spermathecae and ruptured them with forceps into 75 μl of a sperm counting solution. We first created a solution with 10ml spider saline (3.26g NaCl, 0.13g KCl, 0.30g CaCl_2_ + 2H_2_O, 0.26g MgCl_2_ + 6H_2_O and 250ml Distilled Water) and 10 μl of Triton X detergent (solution A). Afterwards, we obtained the sperm counting solution by adding 10ml of spider saline to 150 μl of solution A. We vortexed each sample for 30 s and centrifuged it at 4 x 1000 RPM for 10 min. After repeating this process three times, we placed 10 μl of the supernatant in a counting chamber of a hematocytometer (1mm Neubauer improved). The number of sperm was counted in 16 squares from the 4 corner squares under a microscope 400 x (Olympus VANOX), and the total number was calculated based on the total volume.

### Statistical analyses

Statistical analyses were performed using JMP 7.0 software (SAS institute). Assumptions of parametric tests were examined using Shapiro-Wilk tests for normal distribution of residuals, and Levene’s test for homogeneity of variances. If necessary, data were transformed to meet parametric assumptions. Number of sperm scored in female spermathecae was calculated for both long and short pedipalp insertions. Occurrences of long and short pedipalp insertions in each group were analysed with the Chi-square test. Number of insertions per mating was analyzed using the Mann-Whitney test. Mating duration was analyzed using only data from long insertions, reducing sample size in some analyses (Fly gift-uninterrupted matings, N = 17, Worthless gifts-uninterrupted matings, N = 16; Fly gift-interrupted matings, N = 17; No-fly gift-interrupted matings, N = 13). The relationship between mating duration and sperm stored was analyzed using ANOVA. To analyze the effect of different variables on the amount of sperm stored in the female spermathecae we performed GLM (n) including group, mating duration, type of insertion, male and female sizes. All tests were two-tailed. Raw data are given as supplementary material.

## Results

### Gift content and sperm stored in the female spermathecae

**Fig 2:**
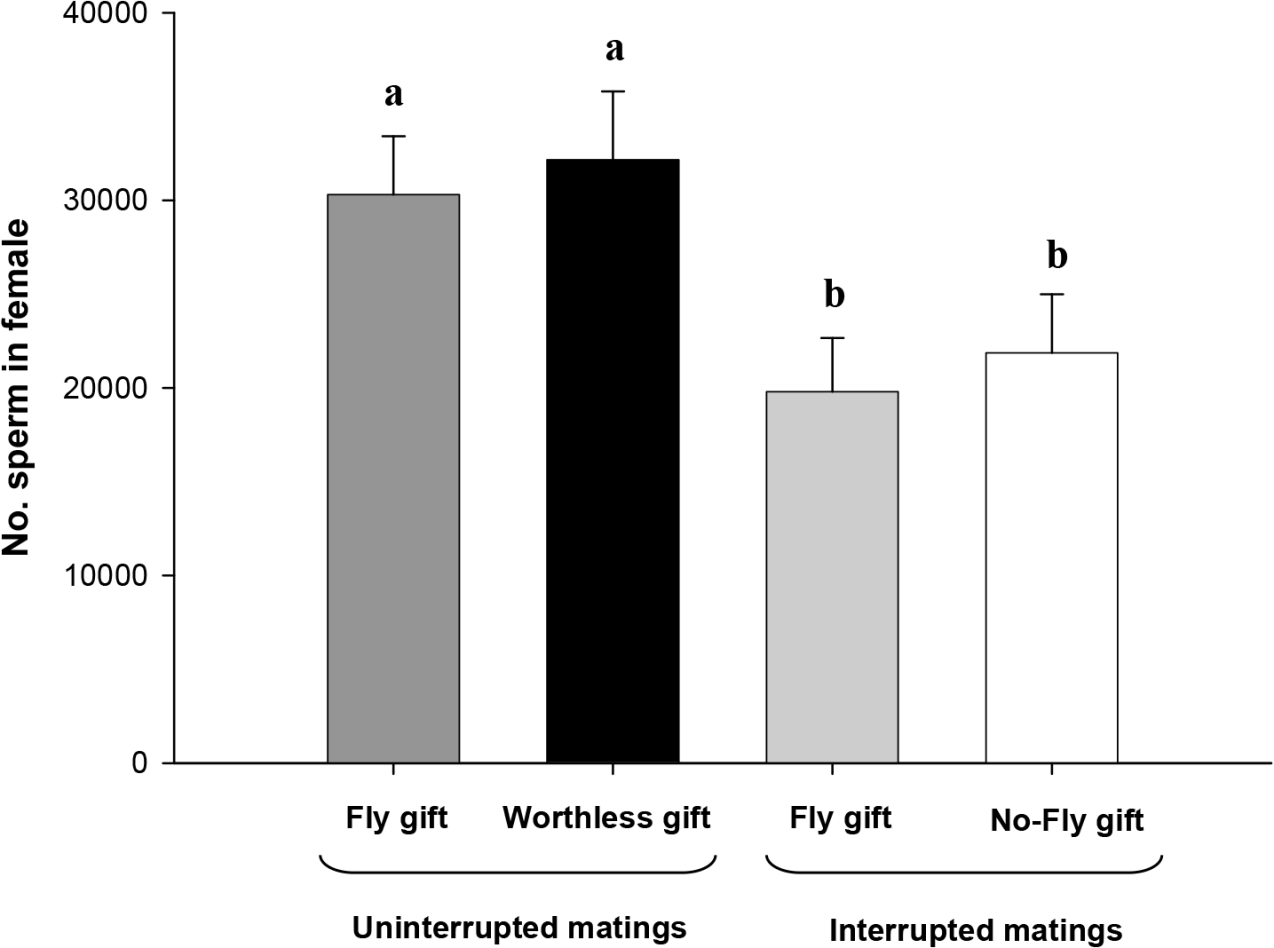
Number of sperm in female spermathecae in uninterrupted matings: Fly gift-uninterrupted mating, males offering a fly gift and Worthless gift-uninterrupted mating, males offering a worthless gift; and in interrupted matings: Fly gift-interrupted mating, males offering a fly gift and performing one pedipalp insertion, No-fly gift-interrupted mating, males offering no gift and performing one pedipalp insertion. Data are shown as Means ± SE; different letters indicate significant differences (p < 0.05; Table 1).

**Fig 3:**
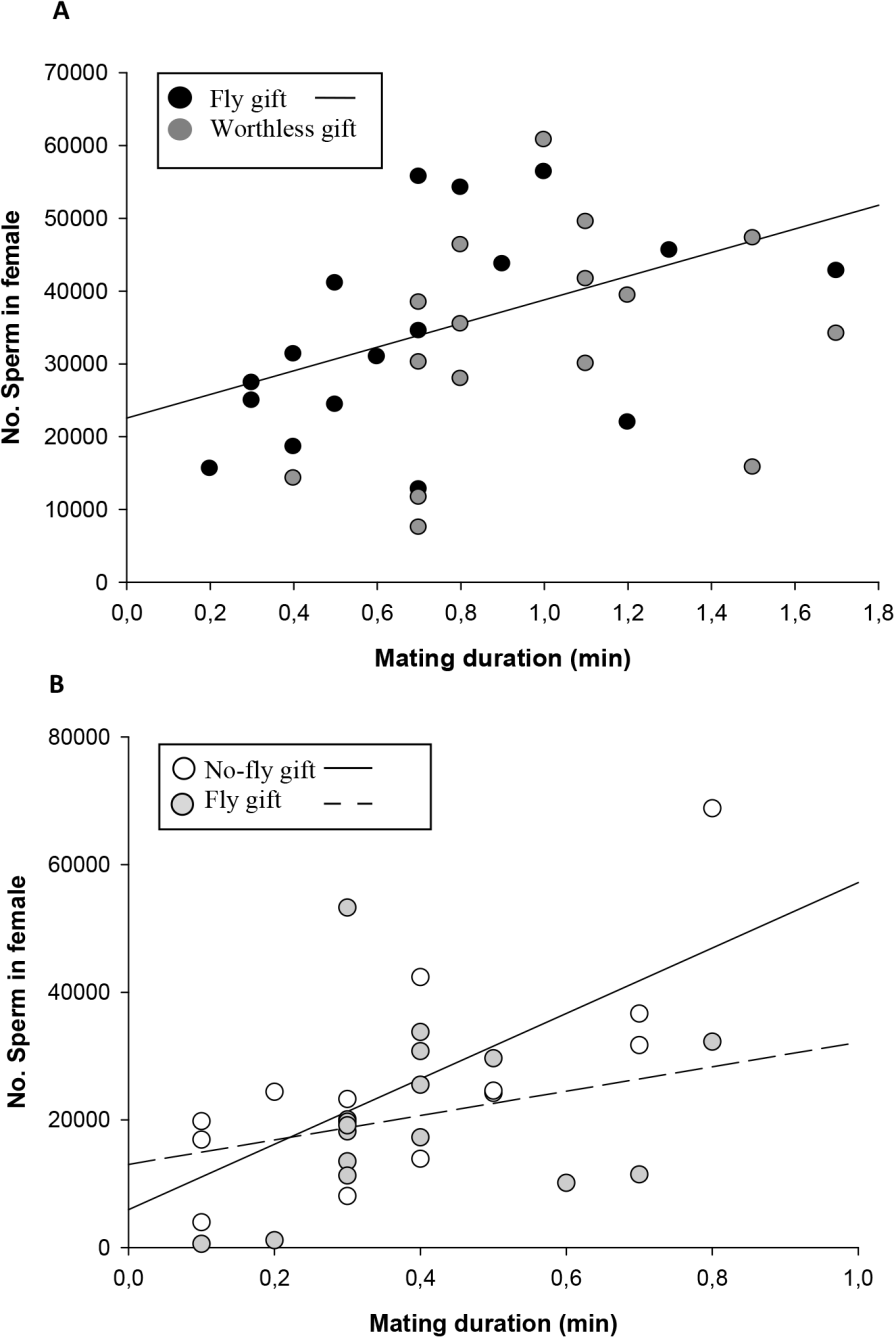
Linear relation between mating duration (min) and number of sperm in female spermathecae in: A) uninterrupted matings: Fly gift-uninterrupted mating, males offering a fly gift and Worthless gift-uninterrupted mating, males offering a worthless gift; B) interrupted matings: Fly gift-interrupted mating, males offering a fly gift and performing one pedipalp insertion, No-fly gift-interrupted mating, males offering no gift and performing one pedipalp insertion. P values are given in Table 1.

Here we varied gift content—worthless and nutritive- and tested whether it affects sperm stored by females. Mating duration averaged 0.72 ± 0.09 min (mean ± SE) in the Fly gift-uninterrupted mating, and 0.99 ± 0.09 min (mean ± SE) in the Worthless gift-uninterrupted mating. In contrast to previous studies [29], mating duration was significantly longer in the Worthless gift-uninterrupted mating group compared to the Fly gift-uninterrupted mating group (GLM: *Χ* ^*2*^_*group*_ = 5.55, p = 0.02), with no effect of individuals’ sizes (GLM: *Χ* ^*2*^_*male size*_ = 0.24, p = 0.62; *Χ* ^*2*^_*female size*_ = 0.02, p = 0.88). Despite this difference, females from Worthless gift-uninterrupted mating group did not store higher numbers of sperm in their spermathecae (Table 1; Fig 2). We found a positive effect of mating duration on the number of sperm in the Fly gift-uninterrupted mating (F_1,15_ = 6.11, p = 0.03; Fig 3A), but not in the Worthless gift-uninterrupted mating (F_1,14_ = 2.87, p = 0.11; Fig 3A). We performed a GLM to examine how several variables together (group, mating duration, type of insertion, male and female size) influence sperm number, and we found that mating duration and type of insertion had a positive effect on the number of sperm (Table 1).

**Table 1:** Effects on the number of sperm stored by females in Fly gift-uninterrupted mating, Worthless gift-uninterrupted mating, Fly gift-interrupted mating, No-fly gift-uninterrupted mating groups.

	Number of sperm in female Fly and Worthless gift-uninterrupted mating	Number of sperm in female Fly and No-fly gift-interrupted mating
**Group (df = 1)**	*Χ* ^*2*^ = 2.67 p = 0.10	*Χ* ^*2*^ = 1.78 p = 0.18
**Mating duration (df = 1)**	*Χ* ^*2*^ = 6.60 p = **0.01**	*Χ* ^*2*^ = 9.92 p = **0.002**
**Type of insertion (df = 1)**	*Χ* ^*2*^ = 6.08 p = **0.02**	-
**Male size (df = 1)**	*Χ* ^*2*^ = 0.49 p = 0.48	*Χ* ^*2*^ = 0.002 p = 0.95
**Female size (df = 1)**	*Χ* ^*2*^ = 2.32 p = 0.13	*Χ* ^*2*^ = 0.05 p = 0.82

The model includes: group, mating duration (min), type of insertion (long/short), male and female sizes (mm). Statistical comparisons were performed using GLM. Significant P values are shown in bold.

**Fig 4:**
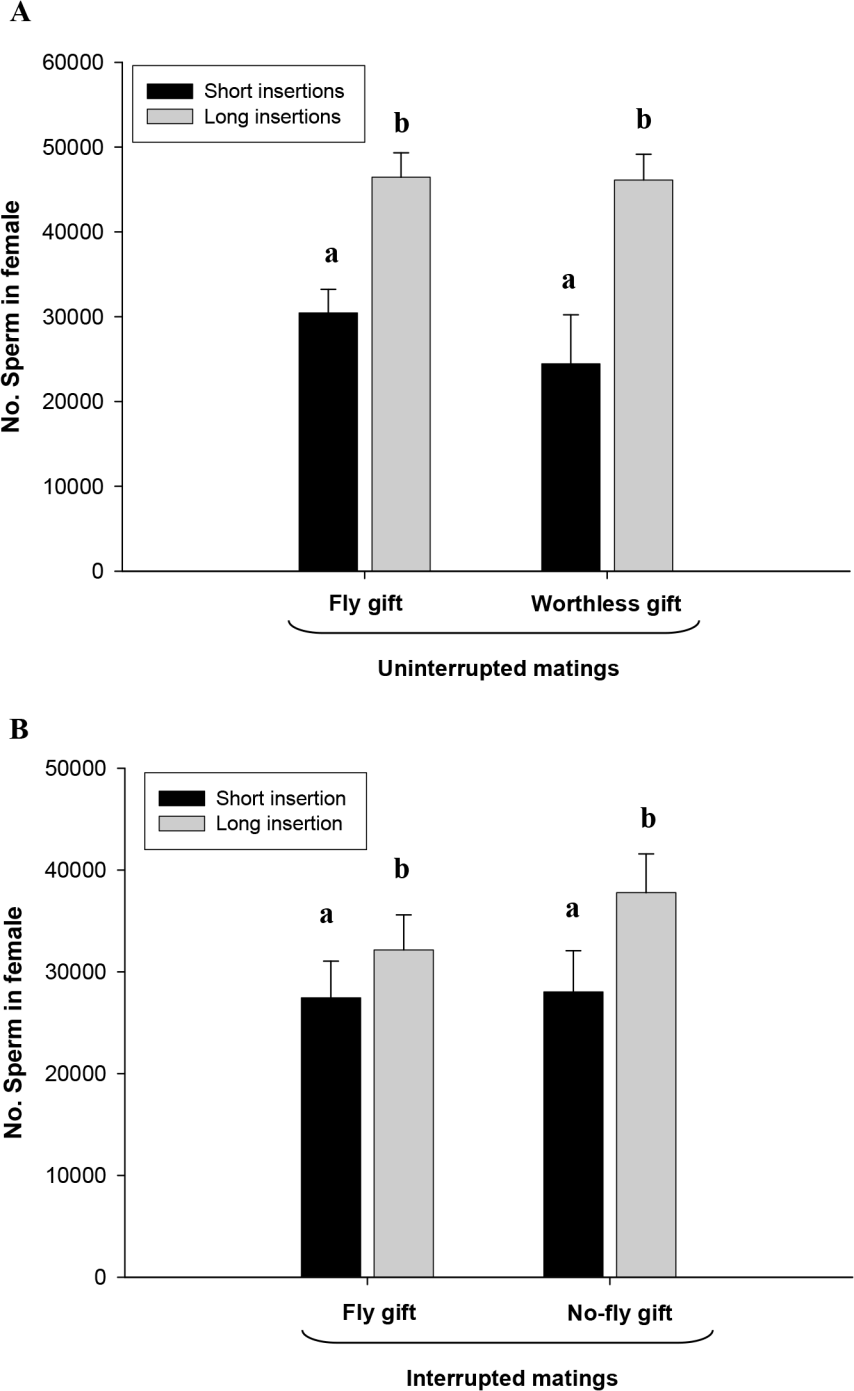
Number of sperm in female spermathecae resulting from long insertions and short insertions in A) uninterrupted matings: Fly gift-uninterrupted mating, males offering a fly gift and Worthless gift-uninterrupted mating, males offering a worthless gift; B) interrupted matings: Fly gift-interrupted mating, males offering a fly gift and performing one pedipalp insertion, No-fly gift-interrupted mating, males offering no gift and performing one pedipalp insertion. Data are shown as Means ± SE; different letters indicate significant differences (p < 0.05; Table 1).

In both Fly and Worthless gift-uninterrupted mating, males often performed long pedipalp insertions while few males performed only short pedipalp insertions (Table 2). Type of insertion did not vary significantly between groups and was not affected by individual sizes (GLM: *Χ* ^*2*^_*group*_ = 1.46, p = 0.23; *Χ* ^*2*^_*male size*_ = 0.30, p = 0.58; *Χ* ^*2*^_*female size*_ = 1.95, p = 0.16). The number of long and short insertions per mating was similar between Fly and Worthless gift-uninterrupted mating (Table 2; Mann-Whitney: long insertions, U = 90.5, P = 0.19, N _Fly gift-uninterrupted mating_ = 16, N _Worthless gift-uninterrupted mating_ = 15; short insertions, U = 4, P = 0.62, N _Fly gift-uninterrupted mating_ = 6, N _Worthless gift-uninterrupted mating_ = 2). Both types of insertions transferred sperm, but in both groups we found more sperm stored in the female spermathecae when males performed long insertions than when males performed only short insertions (Table 1; Fig 4A).

**Table 2:** Occurrence and average number of long and short pedipalp insertions in Fly gift-uninterrupted mating, Worthless gift-uniterrupted mating, Fly gift-interrupted mating, No-fly gift-interrupted mating.

	Fly gift-uninterrupted mating (n = 22)	Worthless gift-uninterrupted mating (n = 17)	Fly gift-interrupted mating (n = 19)	No-fly gift-interrupted mating (n = 24)
**Long pedipalp insertions**				
Occurrence per group	16	15	17	14
Number per mating	1.75 ± 0.15	2.10 ± 0.18	1	1
**Short pedipalp insertions**			
Occurrence per group	6	2	2	10
Number per mating	7.16 ± 2.48	12.5 ± 4.29	3 ± 0	2.3 ± 0.42

Data are shown as Means ± SE.

### Gift presence and sperm stored in the female spermathecae

In this experimental set up we matched copulation time by interrupting matings after the first pedipalp insertion and tested whether gift presence/absence affects sperm stored by females. We found that gift presence did not affect the number of sperm stored in the female spermathecae (Table 1; Fig 2). As expected, females that received one pedipalp insertion (Fly and No-fly gift-interrupted mating) stored significantly lower number of sperm compared to females from uninterrupted matings (Fly and Worthless gift-uninterrupted mating) (ANOVA: F_3,59_ = 12.95, p < 0.0001). We found a positive effect of mating duration on the number of sperm in both groups (Fly gift-interrupted mating: F_1,15_ = 5.08, p = 0.04; No-fly gift-interrupted mating: F_1,11_ = 8.60, p = 0.01; Fig 3B). We performed a GLM to examine how several variables together (group, mating duration, male and female size) influence sperm number, and we found that only mating duration had a positive effect on the number of sperm (Table 1).

In the Fly gift-interrupted mating, males often performed long pedipalp insertions (Table 2); these frequencies differed compared to the No-fly gift-interrupted mating as in this group, a greater number of males performed short pedipalp insertions (Table 2; Chi-square: *Χ* ^*2*^ = 5.11, p = 0.02). This result becomes non significant when male and female sizes are included in a model (GLM: *Χ* ^*2*^_*group*_ = 2.66, p = 0.10; *Χ* ^*2*^_*male size*_ = 3.38, p = 0.06; *Χ* ^*2*^_*female size*_ = 1.78, p = 0.18). In the No-fly gift-interrupted mating group, the number of short insertions ranged between 1–4 (Table 2). In this group, whether the male performed one or more short insertions did not affect the number of sperm in female spermathecae (ANOVA: F_3,6_ = 1.10, p = 0.41). Females stored more sperm when males performed long insertions (GLM: *Χ* ^*2*^_*group*_ = 0.99, p = 0.31, *Χ* ^*2*^_*type of insertion*_ = 5.66, p = 0.02, *Χ* ^*2*^_*male size*_ = 4.90, p = 0.03, *Χ* ^*2*^_*female size*_ = 0.29, p = 0.59; Fig 4B). Also, sperm stored was positive affected by males´ size and apparently matings with smaller males resulted in less sperm stored than those with bigger males.

## Discussion

Our results contribute to the discussion on whether females can discriminate or not between both types of gifts. On one hand, females may prefer males with gifts regardless it content because it represents an attractive and reliable signal. However, it can be also possible that they favour the ones offering nutritive gifts, gaining direct as well as indirect benefits. Here, we expose these two possible evolutionary explanations.

### Nuptial gift reveals male attractiveness or quality

On one hand, the absence of differences in sperm number between females receiving worthless and nutritive gifts could be due to lack of statistical power in the worthless gift group. Indeed, when we analyzed the sperm number in relation to mating duration by pooling both groups (Fly and Worthless gift-uninterrupted mating), the result is a positive and significant relationship. If so and at least under these particular conditions, females do not selectively store more sperm based on gift content, potentially leading to similar paternity success. The origin of male donations must have been due to strong female preferences for nutrients involving fitness benefits for both sexes. Thus, males maximized their own reproductive success offering nutritive gifts, but later they may have reduced costs of gift production resulting in the occurrence of worthless gifts. An invasion of cheating strategy has been suggested as a possible evolutionary path in some gift-giving insects [31, 32]. Due to the high percentage of worthless gift in the field [34], we can suppose that deception reached stability in *P*. *ornata*.

Under this hypothesis, nuptial gift trait has evolved losing its original function as a direct benefit and is now mantained as an indicator of the potential partner. Then, females prefer males with gifts regardless it content because it represents a reliable signal of other male attributes of quality (i.e. better survival or immune response). By choosing gift-giving males, females would benefit from having gift-giving sons that will enjoy higher reproductive success since they will inherit the attractiveness and/or quality from their fathers [25, 38]. Gift presence is indeed an important selected trait during pre-copulatory courtship as females more often accept males with gift (regardless it content) than without gift [33, 34]. Additionally, as has been reported for other gift-giving species [8, 47–50] by using gifts *P*. *ornata* males prolong mating duration [37] and therefore, as it is shown here, they increase the amount of sperm stored by females, potentially increasing paternity success. Further, the gift presence allows males to perform better during mating (probably securing mating position) since they achieve larger number of long insertions compared to males without gift. These last males significantly engage in several short insertions that ultimately would transfer fewer sperm when the mating is completed. Male size may also affect this outcome, although further analyses are needed to understand the potential interaction between both variables. In contrast to *P*. *mirabilis*, in which females select more sperm from males offering gifts than from males without gifts [9], our results from interrupted matings (Fly and No-fly gift-interrupted matings) showed no indication of sperm selection in *P*. *ornata*. Thus, in matings without gift, which are significantly shorter than those with gifts [37], females can simply restrict the number of sperm by shortening the mating.

### Nuptial gift as source of food supply and signal of male donor ability

Alternatively, supposing that there is no lack of statistical power, then, it may be some evidence for female post-copulatory discrimination. Even if males offering worthless gifts were able to mate significantly longer than males offering nutritive gifts, the sperm stored did not increase with mating time, and thus females did not store significantly more sperm in the spermathecae. Males offering worthless gifts may invest heavily in mating duration and achieve longer matings in order to increase sperm stored due to the non-positive relation between mating duration and sperm stored. For instance, several functions are suggested for silk wrapping of the gift, including male control of the gift [51], prolonging mating duration [52] and hiding gift content [33]. We did not control for the amount of silk covering the gifts either with the nutritive or worthless items, but it could be possible that males offering worthless gifts have added more silk than those offering nutritive gifts. Investing in silk wrapping may be a possible strategy for males offering worthless gifts, as according to the suggested functions they would better hide the content and potentially prolong mating duration. Under this scenario, females may be limiting the number of sperm from such males and favour the ones offering nutritive gifts. Beyond the obvious reason of gathering food resources and consequently increasing fecundity (Pandulli I & MJ Albo unpublished data), females can also gain indirect benefits when mating with males offering nutritive gifts [25, 38, 53, 54]. Not surprisingly adult male condition (body weight/cephalothorax width regression) and gift content are linked, as in the field males in better condition usually carry heavier fresh prey while males in lower condition carry lighter empty exoskeletons and/or plant parts [34]. Males in poor condition are likely to consume the prey to gain energy for reproduction [55], while simultaneously reducing gift quality, consequently gift content is a mirror of some aspects of male quality. The possible mechanism used by *P*. *ornata* females to bias sperm stored is unknown. However, they could differentially choose sperm or eject some after mating, as it happens in other species [2, 4, 23, 56]. Sperm dumping have been reported in a wide range of animals, including birds [4], insects [57], and round worms [2, 58]. Nowadays, it is widely accepted that sperm dumping has evolved under sexual selection by cryptic female choice in many species [2, 23, 59]. Otherwise, sperm selection may be regulated via phagocytosis of sperm by hemocytes from the female reproductive tract, recently reported for some arachnids ([60], in solphugids; Peretti AV 2010, pers. obs. in scorpions). However, the importance of this mechanism still requests specific studies in order to confirm its presence and relevance in spiders. Other possibility is that the female influences directly sperm transfer performance, by subtle movements of the body or spermathecae, thus, controlling the entering of the ejaculate from the male’s palp [2].

## Conclusion

In summary, by using nuptial gifts *P*. *ornata* males prolong mating time, consequently as sperm number is commonly positive correlated with mating duration these males would benefit from having more sperm stored by females. Important implications of post-copulatory female control over paternity arise in this mating system where worthless gifts are the rule in the field. Whether females discriminate between males offering worthless and nutritive gifts remains uncertain and much further research is necessary to understand the potential for sexual conflict, including the balance between benefits and costs associated to matings with worthless gifts for both sexes.

## Supporting Information

S1 File File showing the raw data on number of sperm, mating duration and occurrence of long and short pedipalp insertions in A) uninterrupted matings: Fly gift-uninterrupted mating, males offering a fly gift and Worthless gift-uninterrupted mating, males offering a worthless gift; B) interrupted matings: Fly gift-interrupted mating, males offering a fly gift and performing one pedipalp insertion, No-fly gift-interrupted mating, males offering no gift and performing one pedipalp insertion. (PDF)
