# Supplementary material for: Worthless and Nutritive Nuptial Gifts: Mating Duration, Sperm Stored and Potential Female Decisions in Spiders
Source: PLoS One. 2015 Jun 24;10(6):e0129453. doi: 10.1371/journal.pone.0129453 (PMC4480359; doi:10.1371/journal.pone.0129453)
Supplement: S1 File — (PDF) [file pone.0129453.s001.pdf]

(A)

N	Group	Mating duration (min)	1-Long pedipalp insertions 0-short pedipalp insertions	No. sperm stored
1	FG		0	29812,5
2	FG	0,8	1	54187,5
3	FG	1	1	56343,75
4	FG	1,7	1	42750
5	FG	0,7	1	34500
6	FG	0,4	1	31312,5
7	FG	0,2	1	15562,5
8	FG		0	11062,5
9	FG	0,3	1	27375
10	FG		0	15000
11	FG	0,5	1	24375
12	FG	1,3	1	45562,5
13	FG		0	12750
14	FG	0,9	1	43687,5
15	FG	0,5	1	41062,5
16	FG	0,6	1	30937,5
17	FG	0,7	1	12750
18	FG	0,3	1	24937,5
19	FG		0	16500
20	FG	0,7	1	55687,5
21	FG	0,4	0	18562,5
22	FG	1,2	1	21937,5
1	WG		0	16687,5
2	WG	1,5	1	47250
3	WG	0,8	1	35437,5
4	WG	0,7	0	7500
5	WG	0,7	1	30187,5
6	WG	1,2	1	39375
7	WG	0,8	1	46312,5
8	WG	1	1	60750
9	WG	1,1	1	30000
10	WG	0,7	1	38437,5
11	WG	1,1	1	41625
12	WG	1,7	1	34125
13	WG	0,4	1	14250
14	WG	1,5	1	15750
15	WG	0,7	1	11625
16	WG	1,1	1	49500
17	WG	0,8	1	27937,5
**Total**	**N = 39**			

(B)

N	Group	Mating duration (min)	1-Long pedipalp insertions 0-short pedipalp insertions	No. sperm stored
1	FG-1		0	17437,5
2	FG-1	0,3	1	18187,5
3	FG-1	0,3	1	53250
4	FG-1	0,5	1	29625
5	FG-1	0,4	1	25500
6	FG-1	0,3	1	20062,5
7	FG-1	0,3	1	19687,5
8	FG-1	0,8	1	32250
9	FG-1	0,6	1	10125
10	FG-1	0,4	1	33750
11	FG-1	0,4	1	17250
12	FG-1	0,3	1	13500
13	FG-1	0,7	1	11437,5
14	FG-1	0,3	1	19125
15	FG-1	0,4	1	30750
16	FG-1	0,2	1	1125
17	FG-1		0	11250
18	FG-1	0,1	1	562,5
19	FG-1	0,3	1	11250
1	NG-1		0	15937,5
2	NG-1		1	24562,5
3	NG-1	0,8	1	68812,5
4	NG-1		0	48750
5	NG-1	0,4	1	42375
6	NG-1	0,5	1	24187,5
7	NG-1	0,3	1	23250
8	NG-1	0,1	1	16875
9	NG-1	0,7	1	31687,5
10	NG-1	0,5	1	24562,5
11	NG-1	0,4	1	13875
12	NG-1		0	24000
13	NG-1		0	12000
14	NG-1		0	3937,5
15	NG-1		0	8250
16	NG-1	0,7	1	36656,25
17	NG-1	0,3	1	8062,5
18	NG-1		0	16125
19	NG-1	0,1	1	19781,25
20	NG-1		0	16687,5
21	NG-1		0	10312,5
22	NG-1		0	5812,5
23	NG-1	0,2	1	24375
24	NG-1	0,1	1	3937,5

Total **N = 43**
